# Transcriptome profiling of *Issatchenkia orientalis* under ethanol stress

**DOI:** 10.1186/s13568-018-0568-5

**Published:** 2018-03-13

**Authors:** Yingjie Miao, Guotong Xiong, Ruoyun Li, Zufang Wu, Xin Zhang, Peifang Weng

**Affiliations:** Department of Food Science and Engineering, School of Marine Sciences, Ningbo University, Ningbo, 315211 People’s Republic of China

**Keywords:** *Issatchenkia orientalis*, RNA-Seq, Transcriptome, Ethanol stress, Wine fermentation

## Abstract

*Issatchenkia orientalis*, a non-*Saccharomyces* yeast that can resist a wide variety of environmental stresses, has potential use in winemaking and bioethanol production. Little is known about gene expression or the physiology of *I. orientalis* under ethanol stress. In this study, high-throughput RNA sequencing was used to investigate the transcriptome profile of *I. orientalis* in response to ethanol. 502 gene transcripts were differentially expressed, of which 451 were more abundant, and 51 less abundant, in cells subjected to 4 h of ethanol stress (10% v/v). Annotation and statistical analyses suggest that multiple genes involved in ergosterol biosynthesis, trehalose metabolism, and stress response are differentially expressed under these conditions. The up-regulation of molecular chaperones HSP90 and HSP70, and also genes associated with the ubiquitin–proteasome proteolytic pathway suggests that ethanol stress may cause aggregation of misfolded proteins. Finally, ethanol stress in *I. orientalis* appears to have a nitrogen starvation effect, and many genes involved in nutrient uptake were up-regulated.

**Electronic supplementary material:**

The online version of this article (10.1186/s13568-018-0568-5) contains supplementary material, which is available to authorized users.

## Introduction

Fruit wines are fermented alcoholic beverages that derive their flavors from raw materials (fruits, and often flowers and herbs) as well as from the fermentation process. Two distinct yeasts are usually involved in the production of a savory and pleasant fruit wine. The wine yeast *Saccharomyces cerevisiae* is primarily responsible for alcoholic fermentation and the synthesis of secondary metabolites, while non-*Saccharomyces* yeasts or non-conventional wine yeasts contribute additional flavor, texture, and nutritional qualities (Archana et al. 2015). The role of non-*Saccharomyces* wine yeasts in fruit wine fermentation has attracted increasing interest (Ciani et al. 2010). Several studies have focused on multi-strain fermentation and mixed yeast culture (Fleet 2003; Giovani et al. 2012; Sadoudi et al. 2012), and some non-*Saccharomyces* yeasts have been suggested for use in mixed starter cultures with *S. cerevisiae* (Masneufpomarede et al. 2015).

The non-conventional wine yeast *Issatchenkia orientalis* was first described in 1960 but was reclassified to *P*. *kudriavzevii* in 1965 (Kurtzman et al. 2008). Several *I. orientalis* strains produce ethanol and have higher thermotolerance, salt tolerance, and acid tolerance than *S. cerevisiae* (Isono et al. 2012; Koutinas et al. 2016). Because of its resistance to multiple stress factors, *I. orientalis* has potential application in bioethanol production and succinic acid production (Kitagawa et al. 2010; Kwon et al. 2011; Xiao et al. 2014).

High-throughput RNA sequencing (RNA-Seq) is now routinely used to generate global transcription profiles, often to compare gene expression under different conditions. Many studies have used RNA-Seq to examine transcription in *S. cerevisiae* and the fission yeast *Schizosaccharomyces pombe* in response to environmental shifts (Kasavi et al. 2016; Lackner et al. 2012; Lewis et al. 2014). However, gene expression in *I. orientalis* has not yet been studied. In particular, the underlying mechanisms that allow *I. orientalis* to tolerate ethanol have not been explored, nor have they been compared with those in *S. cerevisiae*.

In this study we used RNA-Seq to investigate changes in the gene expression profile of *I. orientalis* under ethanol stress. We identified a wide variety of differentially expressed genes, some of which may play important roles in the stress response.

## Materials and methods

### Yeast strains, media, and growth conditions

*Issatchenkia orientalis* strain CBS 12547 was originally isolated from tropical fruit and food sources, and is involved in the fermentation of some traditional African foods (Greppi et al. 2013; Pedersen et al. 2012). The strain was maintained in the Food Biotechnology Laboratory at Ningbo University. Yeast was initially cultured for 24 h in YPD medium (1% yeast extract, 2% peptone, and 2% glucose) at 30 °C with agitation at 150 rpm. 1 mL was withdrawn, added to 100 mL fresh YPD medium, and incubated as before until the culture reached exponential phase (8 h). For the RNA-Seq experiment, ethanol was added to a final concentration of 10% (v/v), and incubation continued for another 4 h. Three cultures were treated in parallel with ethanol (TE1/TE2/TE3) and three untreated cultures were used as negative controls (T1/T2/T3). Yeast cells were harvested by centrifugation at 4 °C, 2000×*g* and stored at − 80 °C.

### Scanning electron microscopy (SEM)

*Issatchenkia orientalis* was cultured in medium with 10% ethanol for 24 h. Cells were collected by centrifugation at 1000×*g*, 4 °C for 10 min and washed three times with physiological saline. Cells were then resuspended in 2.5% glutaraldehyde for 4 h at 4 °C and washed three times with 0.1 M PBS (pH = 7.4) for 15 min per wash. The cells were transferred through a series of ethanol solutions (30, 50, 70, 80, 90, 95 and 100%; 10 min each), and then through a series of tert-butanol-anhydrous ethanol mixtures (ratio 1:3, 1:1, 3:1, 3:0; 10 min each). Finally, the cells were dried and coated with a gold/palladium alloy (40:60) to a thickness of 10–20 nm and observed with a Hitachi S3400N scanning electron microscopy system.

### Determination of trehalose concentration

*Issatchenkia orientalis* was cultured in medium with 10% ethanol for 0, 4, 12, 24, and 48 h. Cells were collected by centrifugation and washed with ultrapure water. Collected cells were frozen in liquid nitrogen and freeze-dried at − 20 °C. 100 mg of dried cells were resuspended in 1 mL ice-cold 0.5 mol/L trichloroacetic acid solution by brief treatment with ultrasound, and then maintained in the same solution at room temperature for 45 min in order to extract the trehalose from the cells. 250 µL extract was incubated with 1 mL 80% sulfuric acid solution containing 0.2% anthrone in a boiling water bath for 5 min. Absorbance at 620 nm was measured and compared with samples containing known concentrations of trehalose (Sigma-Aldrich) (Kitichantaropas et al. 2016; Mahmud et al. 2009).

### Determination of ergosterol concentration

*Issatchenkia orientalis* was cultured in medium with 10% ethanol for 0, 4, 12, 24, and 48 h. Cells were collected by centrifugation, washed with ultrapure water, then frozen in liquid nitrogen and freeze-dried at − 20 °C. 100 mg of dried cells were resuspended in 3 mL ethanol containing 25% potassium hydroxide (m/v; 25 g KOH dissolved in 35 mL pure water, add ethanol to 100 mL) and incubated at 85 °C for 1 h. The entire sample was mixed with 3 mL *n*-heptane and extracted by vortexing for 3 min. Finally, absorbance of the supernatant at 282 nm was measured and compared with samples containing known concentrations of ergosterol (Sigma-Aldrich) (Arthington-Skaggs et al. 1999).

### RNA extraction, library construction and sequencing

As noted earlier, *I. orientalis* was cultured in medium with 10% ethanol for 4 h before cells were harvested for RNA extraction. Total RNA from each sample was isolated using TRIZOL (Aidlab Biotech, Beijing, China). RNA concentration was quantified using a Qubit^®^ RNA Assay Kit and a Qubit^®^ 2.0 Fluorometer (Life Technologies, CA, USA). RNA integrity and purity were evaluated using the RNA Nano 6000 Assay Kit and the NanoPhotometer^®^ spectrophotometer (IMPLEN, CA, USA).

Construction of cDNA libraries and RNA sequencing were performed by Beijing BioMarker Technologies (Beijing, China). In brief, poly-A mRNA was isolated using poly-T oligomer bound to magnetic beads. mRNA was fragmented using divalent cations at elevated temperature. RNA fragments were copied as cDNA using random primers, and second strand cDNA synthesis was then performed. Double-stranded cDNAs were ligated to a single ‘A’ base and the sequencing adapters. Fragments (200 ± 25 bp) were then separated by agarose gel electrophoresis and selected for PCR amplification as sequencing templates. Finally, the library was constructed for sequencing on the Illumina HiSeq™ 4000 sequencing platform.

### Quality control and read mapping

To obtain high-quality data, raw mRNA-Seq reads were processed using in-house Perl scripts. Reads were discarded if they were spoiled by adaptor contamination, contained ambiguous (N) base calls, or if more than 10% of bases had quality values < 30. The minimum acceptable length was 60 bp to avoid sequencing artifacts. All subsequent analyses were based on the filtered data set. Reads were mapped to the *I. orientalis* reference genome (NCBI Accession Number: GCA_000764455.1) using TopHat2 (http://ccb.jhu.edu/software/tophat/index.shtml). Gene names were assigned to sequences based on matches with the highest score.

### Functional annotation

*Issatchenkia orientalis* genes were aligned to annotated sequences using BLAST (http://blast.ncbi.nlm.nih.gov/Blast.cgi) and the following databases: Nr (NCBI non-redundant protein sequences, Nt (NCBI non-redundant nucleotide sequences, Pfam (Protein family), KOG/COG (Clusters of Orthologous Groups of proteins), Swiss-Prot (manually annotated and reviewed protein sequence database), protein data bank (PDB), KO (KEGG Ortholog database), and GO (Gene Ontology). The Blast2GO suite (Götz et al. 2008) was used to assign GO terms for molecular function, biological process, and cellular component.

### Analysis of differential expression

To compare gene expression level between conditions, the transcript level of each expressed gene was calculated and normalized to fragments per kilobases per million mapped reads (FPKM) using the formula:

$${\text{FPKM}} = \frac{\text{cDNA fragments}}{{{\text{Mapped fragments}}\left( {\text{million}} \right) \times {\text{Transcript Length}}({\text{kb}})}}$$

Differential expression analysis of data from the two experimental conditions (ethanol stress vs. control) was performed using the DESeq R package (1.10.1). *P*-values were adjusted using Benjamini and Hochberg’s approach for controlling the false discovery rate (FDR). Differentially expressed genes (DEGs) were defined as those with fold change > 3 (*P *< 0.05) and FDR < 0.01.

### KEGG and GO enrichment analyses for DEGs

KEGG (http://www.genome.jp/kegg/) is a database resource for understanding functions and utilities of the biological system from molecular-level information, especially large-scale molecular datasets generated by genome sequencing. KEGG is often used to tentatively assign functions and other properties to genes. We used KOBAS (Mao et al. 2005) to determine if any differentially expressed genes were significantly enriched in KEGG pathways. To determine which Gene Ontology (GO) categories were statistically overrepresented among the DEGs, topGO and Cytoscape version 3.4.0 with BiNGO plugin version 3.0.3 (Maere et al. 2005) were used to identify significantly enriched biological networks and to output the results as graphs.

### Quantitative PCR for selected DEGs

Real time quantitative PCR (qPCR) primers for selected DEGs were designed using Primer 5.0 (Additional file 1: Table S1). A Tiangen FastQuant RT Kit (with gDNase) and a KAPA SYBR FAST Universal qPCR Kit were used for reverse transcription and qPCR, respectively. All qPCR reactions were performed using a QuantStudio™ 7 Flex Real-Time PCR system (Applied Biosystems, Thermo Fisher Scientific). The PCR reaction was conducted following the manufacturer’s instructions, and three biological replicates were used in all experiments. Negative controls (template consisting of ultrapure water) were run for each gene. Run-time control of the PCR instrument, baseline correction, and determination of Cq values were performed using QuantStudio™ 7 Flex Real-Time PCR Software v1.2 (Applied Biosystems, Thermo Fisher Scientific).

## Results

### Intracellular trehalose and ergosterol concentration and SEM imaging

**Fig. 1:**
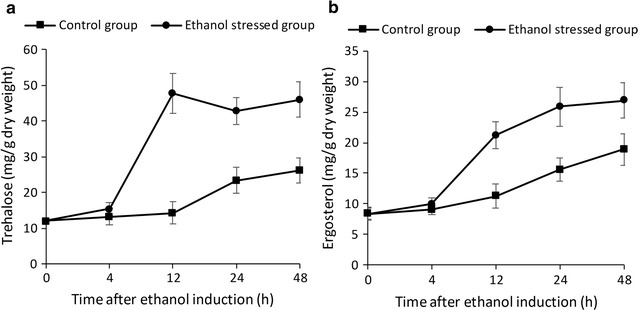
Intracellular concentrations of trehalose and ergosterol in ethanol-stressed *I. orientalis*. The data were obtained at the indicated times (h) after ethanol was introduced into the culture. Values are represented as mean ± S.D. Three biological replicates were used. **a** Intracellular trehalose. **b** Intracellular ergosterol

Intracellular concentrations of trehalose and ergosterol, measured after 4, 12, 24 and 48 h of ethanol stress, are shown in Fig. 1. Compared with unstressed controls, ethanol-stressed yeast cells contained higher levels of both compounds. Carbohydrates such as trehalose and glycogen are compatible solutes that resist osmotic pressure across the cytoplasmic membrane and prevent yeast cells from dehydration. Ergosterol is an important component of the yeast cytoplasmic membrane and is also thought to be involved in stress response.

**Fig. 2:**
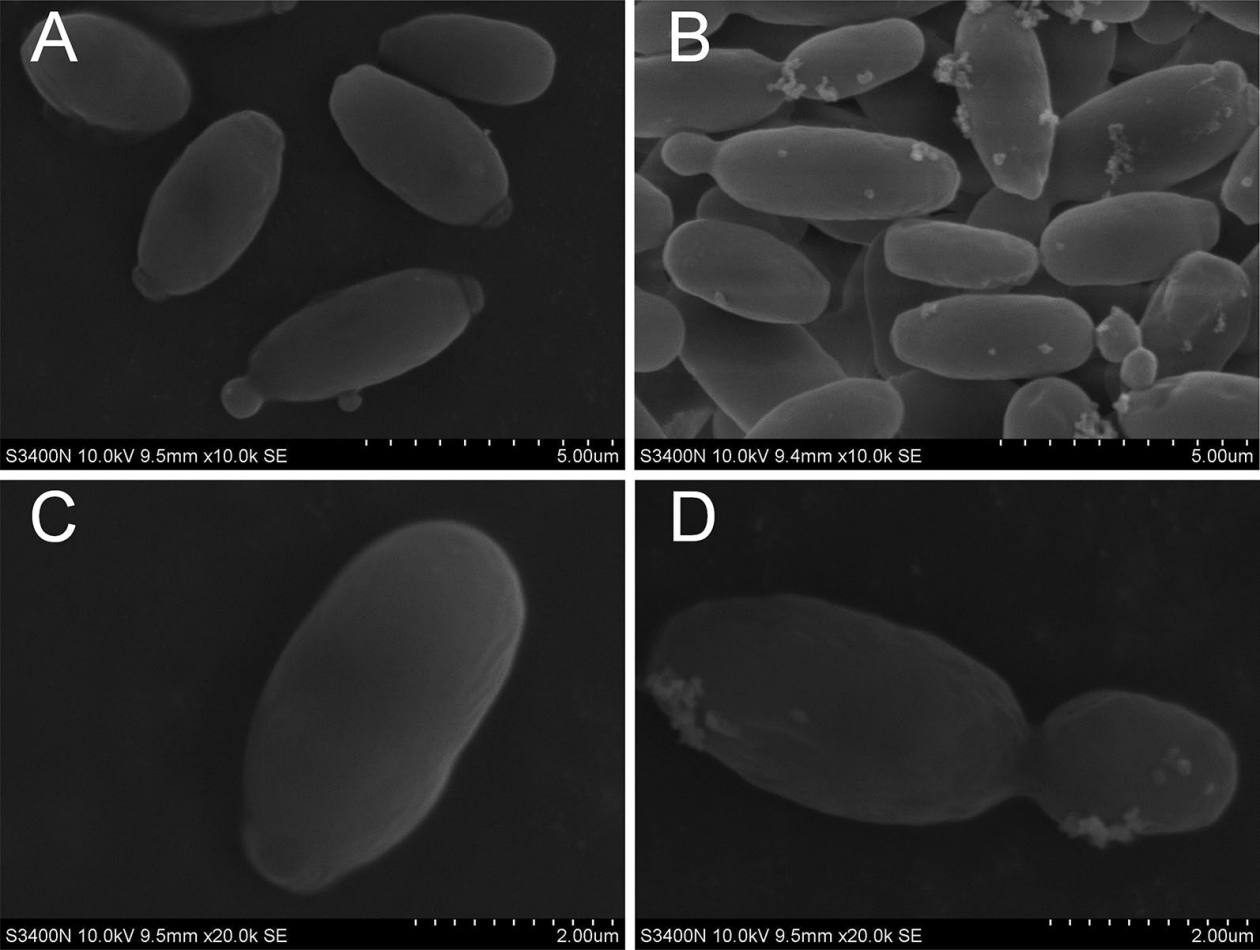
SEM images of control and ethanol-stressed *I. orientalis* cells. SEM images were captured after ethanol stress for 24 h. **A** Control cells at a magnification of 10,000×. **B** Ethanol-stressed cells at a magnification of 10,000×. **C** Control cells at a magnification of 20,000×. **D** Ethanol-stressed cells at a magnification of 20,000×

SEM images were captured after ethanol stress for 24 h (Fig. 2). Stressed cells formed large flocs containing hundreds of connected cells.

### Library construction and RNA-Sequencing

Libraries (NCBI Accession: PRJNA413795) were constructed for RNA-Seq from three control samples (T1-T3; NCBI Accessions: SRX3277329, SRX3277330 and SRX3277331), and three 10% ethanol-stressed samples (TE1-TE3; NCBI Accessions: SRX3277326, SRX3277327 and SRX3277328). After setting aside reads with adaptor contamination, ambiguous base calls, insufficient length, or unacceptable numbers of low quality base scores, 27.15 Gb of high-quality data were obtained with average quality values ≥ 30 for more than 85% of the reads. 76.01% of reads from the control libraries, and 77.17% of reads from the ethanol-stressed libraries, mapped to the *I. orientalis* genome, indicating successful library construction.

### Identification of differentially expressed genes

**Fig. 3:**
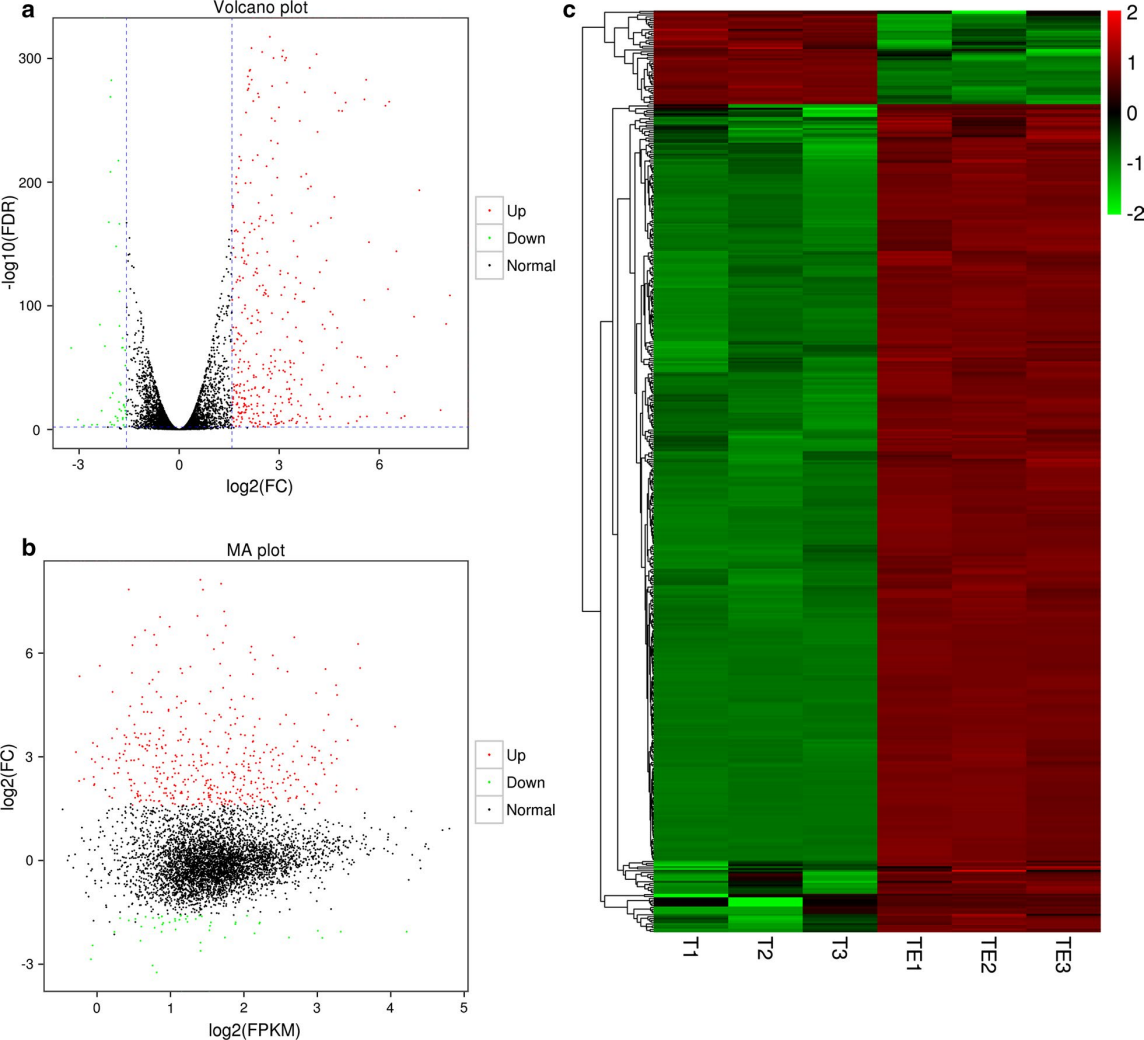
Distribution of DEGs in ethanol-stressed and control *I. orientalis*. **a** Volcano plot showing all DEGs. Dashed lines indicate inclusion criteria for false discovery rate (FDR < 0.01; = line at 2 on the y-axis) and Fold Change (FC > 3; line at ~ 1.6 on the x-axis). Red, more abundant in ethanol-stressed cells; green, more abundant in control cells; black, does not meet inclusion criteria and assumed to be unchanged. **b** MA plot showing FC vs. FPKM for all DEGs. Colors are as in (**a**). **c** Heatmap showing all DEGs. Colors indicate expression levels of DEGs

Gene expression levels were calculated using FPKM values. As shown in Fig. 3, DEGs detected between control and ethanol stressed transcriptomes were required to meet criteria for fold change > 3 (*P* < 0.05) as well as FDR < 0.01. Of 502 transcripts with threefold or greater change, 451 were more abundant and 51 less abundant in ethanol-stressed cells. With four exceptions, all successfully matched with entries in the nr (498), Swiss-Prot (379), KEGG (205), or GO (224) databases, yielding a total of 498 unique and annotated DEGs.

**Fig. 4:**
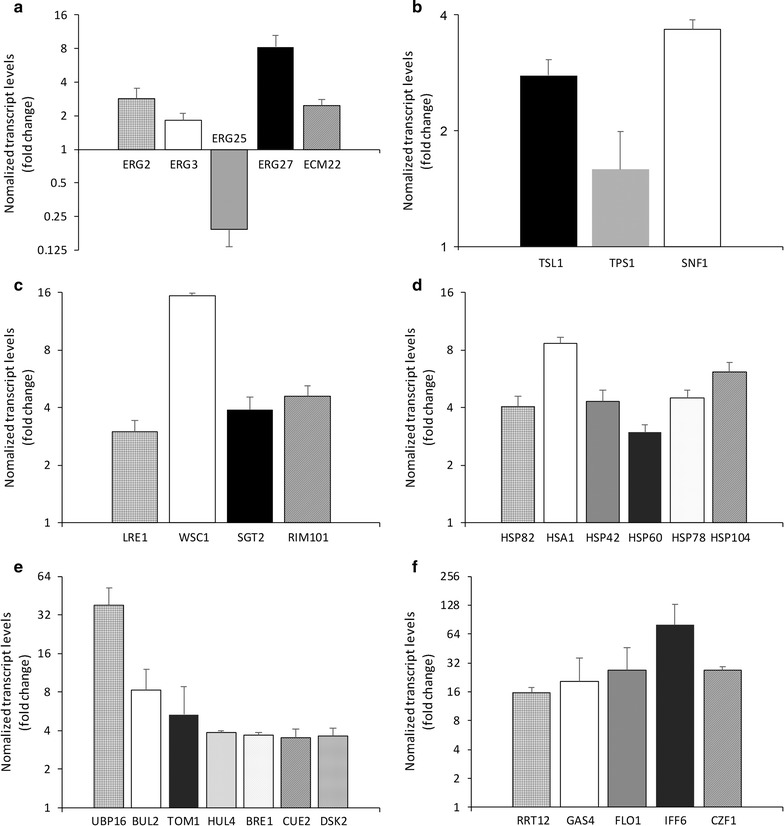
Normalized transcript levels for selected *I. orientalis* DEGs determined by RT-qPCR. Transcript levels (fold changes) for DEGs are shown relative to levels in *I. orientalis* before ethanol stress. Bars represent mean values ± S.D. for three biological replicates. DEGs are grouped by function. **a** DEGs associated with the ergosterol pathway. **b** DEGs associated with the trehalose pathway. **c** DEGs associated with responses to stress and stimulus. **d** DEGs associated with heat shock proteins (HSPs). **e** DEGs associated with the ubiquitin–proteasome proteolytic pathway. **f** DEGs associated with meiosis, sporulation, and ascospore cell wall assembly

Transcript levels for a subset of DEGs in several functional groups were determined by real time quantitative PCR. The results are shown in Fig. 4.

### KEGG pathway analysis

**Fig. 5:**
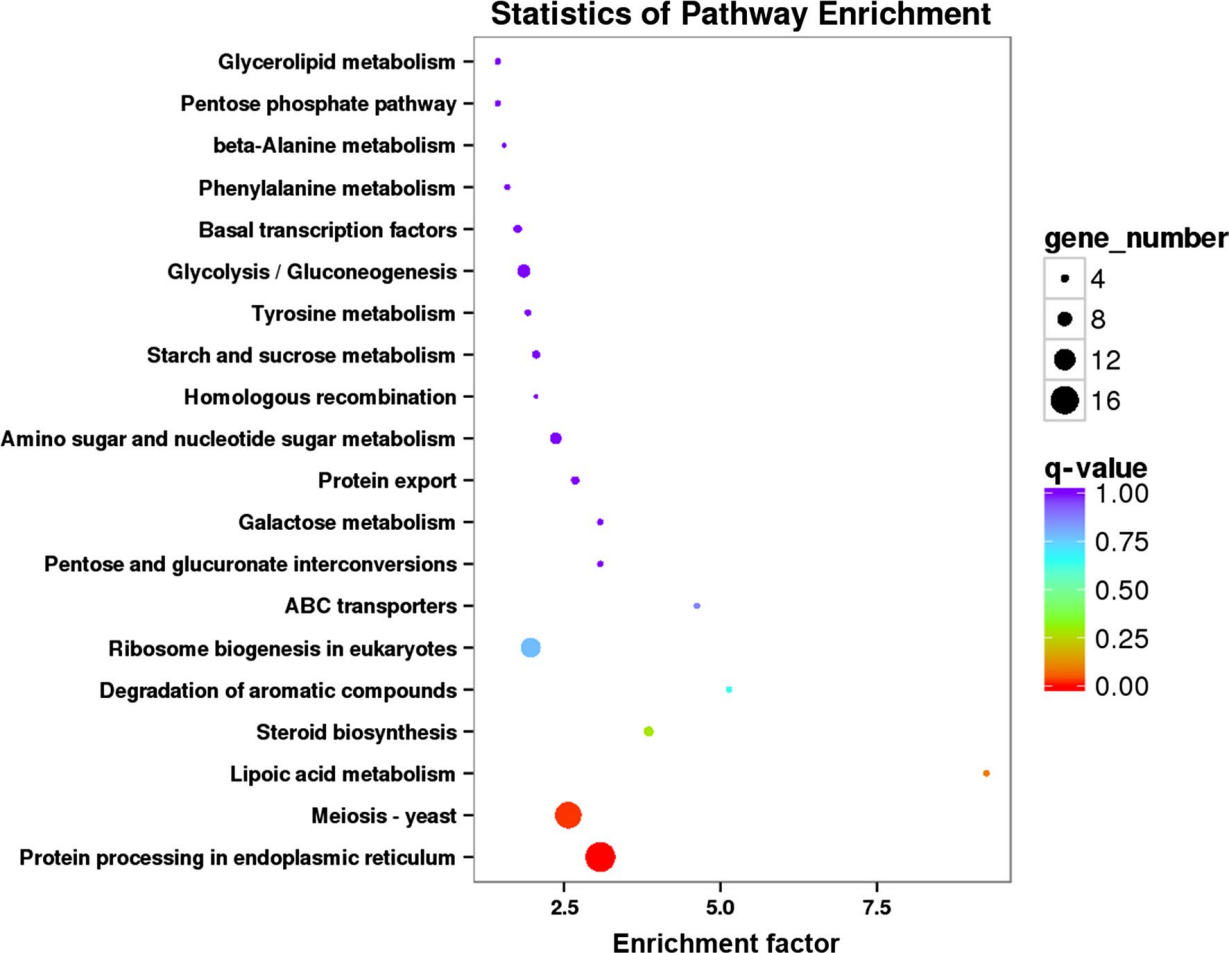
KEGG pathways enrichment analysis. The diameter of the circle is proportional the number of DEGs enriched in each pathway. The color of circle represents the q-value for enrichment

DEGs were annotated using KEGG to identify orthologous genes, and KOBAS was used to test for statistically significant enrichment of DEGs in KEGG pathways. Q-values (Storey 2003) were generated by KOBAS, and are analogous to *P*-values in the context of our analysis. As shown in Fig. 5, DEGs were significantly enriched in pathways used for protein processing in endoplasmic reticulum (ko04141, q < 0.01) and meiosis (ko04113 q < 0.05). Pathways involving lipoic acid metabolism (ko00785) and steroid biosynthesis (ko00100) also had high enrichment scores, but with q values > 0.05.

### GO annotation and analyses

DEGs were annotated and classified by GO category, which are divided into three ontologies: molecular function, cellular component, and biological process. TopGO analysis revealed that several biological process categories (Table 1) were enriched for DEGs, including carbohydrate metabolism, transmembrane transport, ion homeostasis, nuclear or cell division, and process in response to stress or stimuli.

**Table 1:** Enriched biological process terms of the DEGs after ethanol stress (KS < 0.05)

GO:ID	Term	Annotated	DEGs	KS
GO:0005991	Trehalose metabolic process	19	7	0.0018
GO:0048284	Organelle fusion	13	2	0.0025
GO:0015833	Peptide transport	14	7	0.0039
GO:0005978	Glycogen biosynthetic process	7	3	0.0048
GO:0042981	Regulation of apoptotic process	8	2	0.0101
GO:0055085	Transmembrane transport	129	20	0.0143
GO:0005992	Trehalose biosynthetic process	7	4	0.0144
GO:0075136	Response to host	46	6	0.0175
GO:0012501	Programmed cell death	11	2	0.0182
GO:0006875	Cellular metal ion homeostasis	21	4	0.0187
GO:0042173	Regulation of sporulation resulting in formation of a cellular spore	17	3	0.022
GO:0006915	Apoptotic process	9	2	0.0251
GO:0008643	Carbohydrate transport	16	6	0.0262
GO:0044003	Modification by symbiont of host morphology or physiology	38	7	0.0265
GO:0006879	Cellular iron ion homeostasis	10	4	0.0281
GO:0006566	Threonine metabolic process	16	2	0.0326
GO:0006139	Nucleobase-containing compound metabolic process	823	58	0.0334
GO:0005993	Trehalose catabolic process	13	3	0.0357
GO:0043940	Regulation of sexual sporulation resulting in formation of a cellular spore	9	1	0.0358
GO:0040020	Regulation of meiosis	9	1	0.0358
GO:0055082	cellular chemical homeostasis	27	4	0.0397
GO:0006540	glutamate decarboxylation to succinate	7	1	0.0398
GO:0009068	Aspartate family amino acid catabolic process	11	2	0.0429
GO:0046187	Acetaldehyde catabolic process	11	2	0.0429
GO:0006567	Threonine catabolic process	11	2	0.0429
GO:0006117	Acetaldehyde metabolic process	11	2	0.0429
GO:0090304	Nucleic acid metabolic process	621	50	0.0429
GO:0043650	Dicarboxylic acid biosynthetic process	6	1	0.0435
GO:0006457	protein folding	38	10	0.0439
GO:0030003	Cellular cation homeostasis	25	4	0.0453
GO:0051701	Interaction with host	88	11	0.0454
GO:0052173	Response to defenses of other organism involved in symbiotic interaction	56	6	0.0468
GO:0031349	Positive regulation of defense response	26	6	0.047
GO:0052510	Positive regulation by organism of defense response of other organism involved in symbiotic interaction	26	6	0.047
GO:2000241	Regulation of reproductive process	13	1	0.0476

**Fig. 6:**
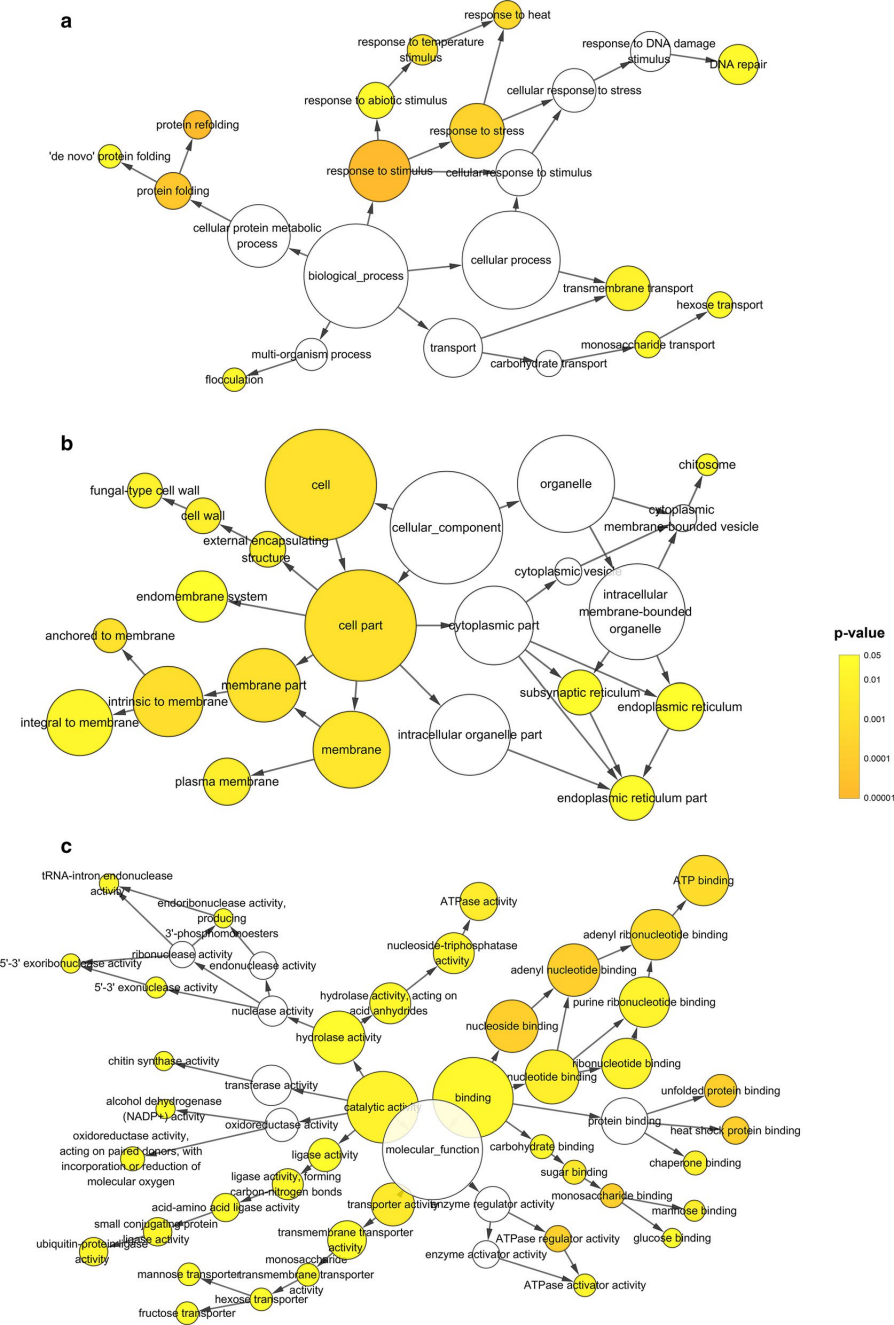
Gene Ontology enrichment analysis using Cytoscape-BiNGO. The number of enriched DEGs in each GO category is proportional to node diameter. Darker nodes are associated with lower *P*-values. **a** Biological process. **b** Cellular component. **c** Molecular function

Figure 6 shows molecular interaction graphs for the three GO ontology classifiers, generated using Cytoscape-BiNGO. In the biological process ontology (Fig. 6a), statistically overrepresented GO categories can be divided into five groups (process in response to stimuli, protein folding and refolding, sugar transport, DNA repair and flocculation). In the molecular function ontology (Fig. 6b), overrepresented GO categories can be divided into four clusters. The largest group consists of binding functions, specifically nucleotide binding, protein binding, and sugar binding. Other groups involved ATP hydrolase activities, ubiquitin protein ligase activities, and sugar transmembrane transport activities. In the cellular component ontology (Fig. 6c), the overrepresented GO categories included cell wall, ER, and plasmid membrane.

A protein–protein interaction (PPI) network was generated to identify key proteins involved in the response made by *I. orientalis* to ethanol stress (Additional file 2: Figure S1). Five proteins in this network (DSK2, HSP82, HSA1, BiP, and SMK1) are significantly and differentially expressed. These may play important roles in the stress response.

## Discussion

*Issatchenkia orientalis*, a non-*Saccharomyces* yeast that can tolerate a variety of stressful environments, is potentially useful in winemaking and bioethanol production. However, it is less tolerant to ethanol than *S. cerevisiae* (Archana et al. 2015), and can grow and ferment only when ethanol concentrations are under 10%. In *S. cerevisiae*, a cluster of environmental stress response (ESR) family genes have coordinated expression under a variety of stress conditions (Gasch et al. 2001), and 73 genes in the ESR family are up-regulated during ethanol stress (Alexandre et al. 2001). In contrast, little is known about gene and protein expression in *I. orientalis* under environmental stress. In this study, RNA-Seq was used to conduct a genome-wide transcriptional survey of *I. orientalis* during a short period of ethanol stress (4 h). 502 genes were identified as differentially expressed under these conditions. Among these, 451 and 51 genes were up-regulated and down-regulated, respectively, with fold change > 3 (*P *< 0.05) and FDR <  0.01.

### Ergosterol biosynthesis

KEGG enrichment analysis identified the steroid biosynthesis pathway (ko00100) as highly enriched (Fig. 5) including many DEGs associated with steroid biosynthesis (especially ergosterol biosynthesis). In *S. cerevisiae*, ergosterol protects cell membrane integrity and enhances membrane fluidity in response to stress (Chi and Arneborg 2000; Ren et al. 2014), but genes associated with ergosterol biosynthesis are transcriptionally down-regulated (Alexandre et al. 2001).

We found that ergosterol accumulates after ethanol stress (Fig. 1). Transcripts for the ergosterol biosynthesis genes *ERG2*, *ERG3,* and *ERG27* are significantly more abundant in ethanol-stressed cells, in contrast to results reported for these genes in *S. cerevisiae*. *ECM22*, which encodes a sterol element-binding transcription factor that regulates sterol uptake and sterol biosynthesis (Woods and Höfken 2016), is also more abundant. *ERG25* is an exception, and is less abundant under ethanol stress. The results confirm the role of ergosterol in *I. orientalis* as an important cytoplasmic membrane protectant in response to ethanol stress.

### Trehalose metabolism

Analyses (Table 1, Fig. 4) show that genes involved in trehalose and glycogen metabolism are up-regulated during ethanol stress. The intracellular carbohydrates trehalose and glycogen are compatible solutes that resist osmotic pressure across the cytoplasmic membrane. Trehalose is involved in ethanol tolerance in *S. cerevisiae* (Mahmud et al. 2009; Wang et al. 2013; Yi et al. 2016). The up-regulation of trehalose and glycogen synthesis genes, and the accumulation of trehalose (Fig. 1), are consistent with this role. Stress tolerance in yeast may rely on trehalose-6p synthase (TPS1), the first enzyme in trehalose biosynthetic pathway, rather than on trehalose itself (Petitjean et al. 2015). In fact, we found that several genes in trehalose biosynthetic pathway, including *TPS1*, are up-regulated during ethanol stress. We conclude that the regulation of the trehalose pathway plays an important role in protecting cells against ethanol stress in *I. orientalis.*

### Response to stress and stimulus

Genes involved in the response to biotic and abiotic stimulus, including heat and pH, were also enriched (Table 1, Fig. 6). Up-regulation of heat stress response genes, such as *LRE1*, *WSC1*, *SGT2*, and a variety of heat shock proteins, was observed in all samples in response to ethanol. In stress-tolerant *S. cerevisiae* strains, intracellular trehalose accumulates and heat shock protein genes are continuously induced in response to stresses that damage proteins, including heat, ethanol, osmotic, and oxidative stress (Kitichantaropas et al. 2016).

Expression of *RIM101*, a pH-response transcription factor, was up-regulated in response to ethanol. The homologous gene in *S. cerevisiae* regulates response and resistance to low pH and acidic conditions (Mira et al. 2009). In *S. cerevisiae,* high concentrations of ethanol affect the integrity of the cell membrane, changing proton permeability and causing intracellular acidification (Rosa and Sá-Correia 1996; Teixeira et al. 2009). Vacuolar acidification is a potential mechanism to recover cytosolic homeostasis after ethanol-induced intracellular acidification in *S. cerevisiae* (Martínez-Muñoz and Kane 2008). Similar mechanisms in *I. orientalis* may help *I. orientalis* maintain pH stability in the presence of ethanol.

### HSP90, HSP70, and ubiquitin

Genes associated with protein folding and refolding (Fig. 6) are up-regulated under ethanol stress, such as *HSP42*, *HSP78*, and *HSP104* (Fig. 4). PPI analysis suggests an important role for *HSP82* (homolog of yeast *HSP90*) and *HSA1* (*HSP70* 1) in protein folding and refolding (Additional file 2: Figure S1). Based on our RNA-Seq results, other genes encoding HSP binding proteins and co-chaperones such as *STI1*, *AHA1*, *SSE1*, *MAS5*, *FES1*, and *SIS1* are also up-regulated.

In eukaryotes, HSP90 proteins are conserved, abundant molecular chaperones involved in many essential cellular processes (Li et al. 2012). Two cytosolic HSP90 isoforms exist in yeast: an inducible form HSP82, and a constitutive form HSC82. The association of HSP90 with HSP70 and a variety of co-chaperones generates large dynamic multi-chaperone complexes known as HSP90/HSP70 machinery. These play critical roles in the recruitment and assembly of client proteins, and also work in concert with the ubiquitin–proteasome system (UPS), directing misfolded proteins for degradation (Li et al. 2012). HSP42, HSP78, and HSP104, which were mentioned above, also help process aggregations of unfolded or misfolded proteins (Glover and Lindquist 1998).

Cytoscape-BiNGO analysis suggests that proteins with ubiquitin-protein ligase activity are up-regulated, including genes encoding ubiquitin-associated proteins (*UBP16*, *BUL2*, *TOM1*, *HUL4*, *BRE1*, and *CUE2*). The UPS degrades proteins that have exceeded their functional lifetime and destroys most unfolded and misfolded proteins (Amm et al. 2014). Proteins with ubiquitin-protein ligase activity, mainly E3 ligases, often work with HSP90/HSP70 chaperone systems and recognize misfolded proteins (Berndsen and Wolberger 2014; Petrucelli et al. 2004). The gene encoding ubiquitin domain-containing protein DSK2, which involved in the ubiquitin–proteasome proteolytic pathway and in spindle pole body duplication, was identified by PPI analysis as a key factor in the response to ethanol stress (Additional file 2: Figure S1).

The up-regulation of genes encoding HSP proteins and E3 ubiquitin ligases suggests that protein misfolding occurs under ethanol stress, possibly affecting proteins that help maintain plasma membrane integrity and function. Since the accumulation of improperly folded proteins is toxic, the HSP90/HSP70 based chaperone machinery and the ubiquitin–proteasome proteolytic pathway may be essential in the response to ethanol stress.

### Starvation effect and transport

Genes associated with meiosis, reproduction, sporulation, ascospore cell wall assembly, and membrane biogenesis were up-regulated (Fig. 6, Table 1). For example, *RRT12* encodes a spore wall-localized subtilisin-family protease required for spore wall assembly (Suda et al. 2009). *GAS4* encodes a 1,3-beta-glucanosyltransferase that elongates 1,3-beta-glucan chains during spore wall assembly (Ragni et al. 2007). *FLO1* encodes a cell wall protein that participates directly in adhesive cell–cell interactions during yeast flocculation (Fichtner et al. 2007). *IFF6* encodes a GPI-anchored cell wall protein involved in cell wall organization and hyphal growth. Finally, CZF1 is a transcription factor involved in the regulation of filamentous growth in yeasts that responds to temperature and carbon source (Brown et al. 1999; Vinces et al. 2006). It is possible that CZF1 is involved in the flocculation of *I. orientalis* cells that we observed under ethanol stress (Fig. 2).

Genes with transporter activities were also up-regulated. These include genes involved in amino acid and peptide transport (transporter specific for methionine, cysteine and oligopeptide), carbohydrate transport (transporter specific for hexose such as mannose, fructose and glucose) and transmembrane transport. In addition, genes involved in protein transport, coenzyme transport, lipid transport, a-factor pheromone transport, and genes in the major transporter facilitator superfamily (MFS) were up-regulated.

Nitrogen starvation in *S. cerevisiae* induces meiosis, pseudohyphal growth, and sporulation. The presence of ethanol may affect the transmembrane transport of nutrients, leading to a pseudo-starvation state that elicits a nitrogen starvation response by the cell (Chandler et al. 2004; Kasavi et al. 2016; Stanley et al. 2010). Consistent with this hypothesis, up-regulation of meiosis, sporulation, and transportation-associated genes suggests that *I. orientalis* responds to ethanol stress as if it were experiencing nitrogen starvation. In effect, *I. orientalis* cells mistakenly perceive that they are growing in a nutrient-deficient environment, rather than in a nutrient-complete culture medium. The up-regulation of transmembrane transport genes is thus an attempt by the cell to cope with the pseudo-starvation state caused by ethanol stress.

The pseudo-starvation state may be due to the lack of coenzymes such as NAD + and coenzyme A (CoA). NAD + is an important cofactor for the glycolysis enzyme glyceraldehyde 3-phosphate dehydrogenase (GAPDH), while CoA is required for fatty acid metabolism and the oxidation of pyruvate in the citric acid cycle. We found that several genes encoding NAD(P) + -dependent enzymes were up-regulated, which implies that demand for NAD(P) + had increased. This is consistent with the transcriptional activation of *Liz1* (Stolz et al. 2004), which encodes a plasma membrane-localized transport protein for the uptake of pantothenate, the precursor of coenzyme A (CoA). A lack of pantothenate would result in slow growth, delayed septation, and mitotic defects.

In conclusion, our data provide a global view of transcriptional changes in *I. orientalis* under ethanol stress. The changes are likely to reflect adaptation to stressful conditions at multiple levels. We observed modifications in the trehalose and ergosterol biosynthetic pathways, and also activation of various genes related to stress. Examples include heat shock proteins and their co-chaperones, which refold aggregated and misfolded proteins, and the ubiquitin–proteasome system, which targets misfolded proteins for degradation. Finally, ethanol stress appears to induce a nutrition starvation effect, which is associated with changes in cellular uptake, pseudohyphal growth, and sporulation. These results provide a basis for future investigations of the mechanisms that regulate ethanol stress in *I. orientalis*.

## Additional files

**Additional file 1: Table S1.** RT-qPCR Primers used in this study.

**Additional file 2: Figure S1.** Protein–Protein Interaction network.

## Abbreviations

DEGs: differentially expressed genes
FC: fold change
FDR: false discovery rate
GO: gene ontology
NGS: next generation sequencing
PDB: protein data bank
PIR: protein information resource
PPI: protein–protein interaction
PRF: Protein Research Foundation
RNA-seq: RNA-sequencing
FPKM: fragments per kilobases per million mapped reads
RT-qPCR: reverse transcription quantitative PCR
SEM: scanning electron microscopy
