# Supplementary material for: Transcriptome profiling of Issatchenkia orientalis under ethanol stress
Source: AMB Express. 2018 Mar 13;8:39. doi: 10.1186/s13568-018-0568-5 (PMC5849708; doi:10.1186/s13568-018-0568-5)
Supplement: Supplementary file 1 — Additional file 1: Table S1. RT-qPCR Primers used in this study. [file 13568_2018_568_MOESM1_ESM.pdf]

- Journal name:

AMB Express

- Manuscript Title:

Transcriptome Profiling of *Issatchenkia orientalis* under Ethanol Stress

- The name(s) of the author(s):

Yingjie Miao

Guotong Xiong

Ruoyun Li

Zufang Wu

Xin Zhang

Peifang Weng

- The affiliation(s) and address(es) of the author(s):

Department of Food Science and Engineering, School of Marine Sciences, Ningbo University

No.818, Fenghua Rd., Ningbo, Zhejiang, P.R. China, 315211

- The e-mail address, telephone and fax numbers of the corresponding author:

Corresponding Author: Zufang Wu

E-mail: wzfwpf@163.com

Telephone: 086-0574-87600551

Fax: 086-0574-87608347

Table S1 RT-qPCR Primers used in this study.

Gene Name	F-primer sequence (5'-3')	R-primer sequence (5'-3')	product size (bp)
ERG2	ATATGGCGAAGGGCGAGTTC	CCAGCGTACTGGCAAAGGTA	126
ERG3	CATATGCAACCCTGCTCCCA	TGAATAGTGCCACAACAACAACT	104
ERG25	TGGAAAGAGTATGGGCTGCAT	GCTTGGGTAATTGTTTGGTTGGA	176
ERG27	CCCAATCAAAGGTAGTTGGTGTG	TGGATGAAATAGTAAGGGCCGA	88
ECM22	ATGGTCCACCACCACCATAC	CTGGGCTGCTGATTTAGCCA	107
TSL1	CCTTGGAGATGAGGCACCTG	ACACCATCTTCCGTCGAGGT	117
TPS1	CTGGTGCTGCCCAATCTTTG	TGCATCTAGAGAGTTCTTTGACA	198
SNF1	TCAGCAAGCAAGATCTAAGAAAGT	AGGCTTTAAGTCTCGGTGGAC	88
LRE1	TACGTGTGCTCCCTCGTTAG	TAGCGGATGTGGTAGGTGTG	110
wsc1	ACGGAGACTGAAACGACCAC	AGCTGCAGTAACTCATCTTCAT	140
SGT2	CAGAAGCTTTCGCCGTTGAG	CCAAAGAAGGTCGGACTCGT	173
RIM101	GAAGAAGTGAACAGCACCGC	TAACACTTGGGCATGCGACT	110
HSP82	GCCATTGTGGACTAGAAACCC	ATGGCGCTCTCTTTGGAACA	164
HSA1	TCACCGACACTGAGAGGTTG	TGCACTTCTGCATCATTGAACT	121
HSP42	GGGGTGGACGATTCACAGAT	GCTCTCCCGGAGGTTAAGAAG	147
HSP60	AACGCTGGTGGTGAAGGATG	GCTACACCAGCTGCATCAAC	173
HSP78	GAGGGATTGGCGCAGAGAAT	CCCCTGTATTTGGCACCACT	110
hsp104	GCGGGCAATGATCAGAATCG	TAAGTTTTGGACGGGTGCAG	187
UBP16	AAACGCTGACTGACGTATGG	CTGCAAGTAACGATGAGCCC	123
BUL2	GCAGCCACCACTGTACCTT	CTTATCGTCAATTGGCCGGTTT	100
TOM1	ACTGCTACTACTGCGGACTC	GCCATGGTCATTGCTCTCAC	148
HUL4	CCGGGGAAGAAGAGGGAGAA	AAGGAGCGTTCCACAACAGG	141
BRE1	CACCATGCATCACCGTTTGAA	AGGCCCGTCACTGGAAATG	102
CUE2	AATGTTCAGCATGTTCCGCCC	CCAGTCACTTGAATGCTAGAACG	152
DSk2	ACACGGATTCGCCACGATTA	AAACCTGCTCCAGGAACACC	103
RRT12	CTTCCTAAACTTGGACTTAAAAGGT	GCCGCACTAAATGAGAGGGT	145
gAS4	TCTTTCCGATATGAGGCAGATTCC	TTGTTCTCCACACCACTGATAC	157
FLO1	CTTCTTTATCGTCTGCGCCA	AGTTGGTGAGCAGTCAGTGG	149
IFF6	TTTCCACCTCCATTACCGCC	ATGGTGGTAGTTGCATGGGT	100
CZF1	TGGAGAGTAATTCAGACAACAGGA	GGCAGTGGCCATCTACACTT	145
