# Supplementary material for: Transcriptome profiling of Issatchenkia orientalis under ethanol stress
Source: AMB Express. 2018 Mar 13;8:39. doi: 10.1186/s13568-018-0568-5 (PMC5849708; doi:10.1186/s13568-018-0568-5)
Supplement: Supplementary file 2 — Additional file 2: Figure S1. Protein–Protein Interaction network. [file 13568_2018_568_MOESM2_ESM.pdf]

- Journal name:

AMB Express

- Manuscript Title:

Transcriptome Profiling of *Issatchenkia orientalis* under Ethanol Stress

- The name(s) of the author(s):

Yingjie Miao

Guotong Xiong

Ruoyun Li

Zufang Wu

Xin Zhang

Peifang Weng

- The affiliation(s) and address(es) of the author(s):

Department of Food Science and Engineering, School of Marine Sciences, Ningbo University

No.818, Fenghua Rd., Ningbo, Zhejiang, P.R. China, 315211

- The e-mail address, telephone and fax numbers of the corresponding author:

Corresponding Author: Zufang Wu

E-mail: wzfwpf@163.com

Telephone: 086-0574-87600551

Fax: 086-0574-87608347

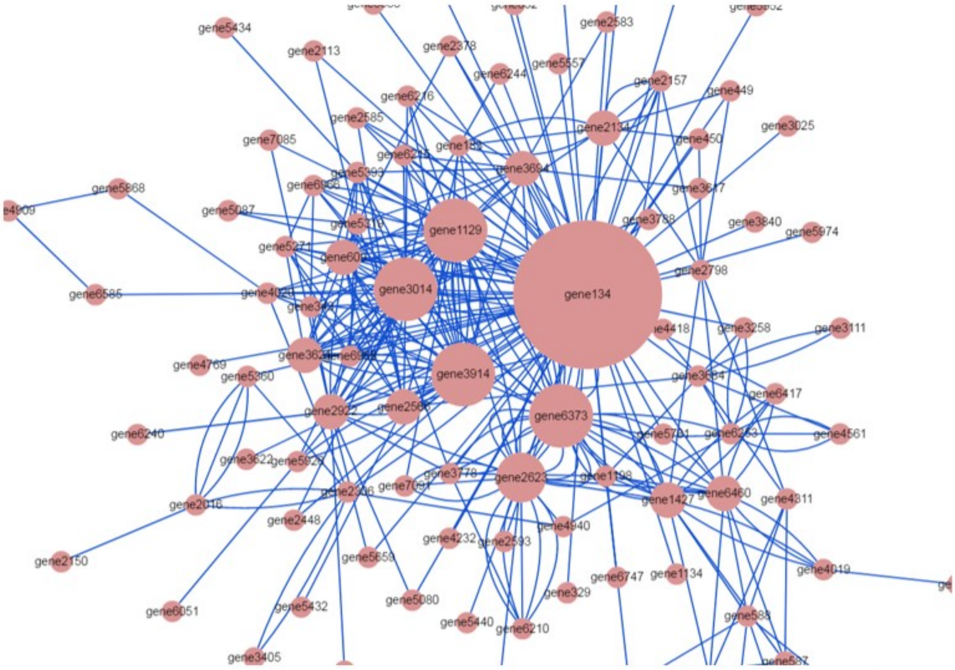

Unigene ID	Gene Name	Decription
gene134	DSK2	Ubiquitin domain-containing protein DSK2
gene1129	HSP82	Heat shock protein 90 homolog
gene3014	HSA1	Heat shock protein 70 1
gene3914	BiP	78 kDa glucose-regulated protein homolog (Precursor)
gene2623	SMK1	Sporulation-specific mitogen-activated protein kinase SMK1

Fig. S1 Protein-Protein Interaction network.

The diameter of the circle represents the number of interactions with neighbor proteins.
